# Full-Length Transcriptome Sequencing and Different Chemotype Expression Profile Analysis of Genes Related to Monoterpenoid Biosynthesis in *Cinnamomum porrectum*

**DOI:** 10.3390/ijms20246230

**Published:** 2019-12-10

**Authors:** Fengying Qiu, Xindong Wang, Yongjie Zheng, Hongming Wang, Xinliang Liu, Xiaohua Su

**Affiliations:** 1 State Key Laboratory of Tree Genetics and Breeding, Research Institute of Forestry, Chinese Academy of Forestry, Beijing 100091, China; qiufengying1@163.com; 2 Jiangxi Academy of Forestry, Camphor Engineering Technology Research Center for National Forestry and Grassland Administration, Nanchang 30032, China; x_wangxindong@sina.com (X.W.); zyj920581676@gmail.com (Y.Z.); liuxinliang1988@163.com(X.L.); 3 College of Bioengineering and Biotechnology, Tianshui Normal University, Tianshui 741000, China; hongmw26@163.com; 4 Key Laboratory of Tree Breeding and Cultivation, National Forestry and Grassland Administration, Beijing 100091, China

**Keywords:** *Cinnamomum porrectum*, terpenoids, terpenoid biosynthesis, full-length transcriptome, different chemotypes

## Abstract

Leaves of *C. porrectum* are rich in essential oils containing monoterpenes, sesquiterpenes and aromatic compounds, but the molecular mechanism of terpenoid biosynthesis in *C. porrectum* is still unclear. In this paper, the differences in the contents and compositions of terpenoids among three chemotypes were analyzed using gas chromatography mass spectrometry (GC/MS). Furthermore, the differential expression of gene transcripts in the leaf tissues of the three *C. porrectum* chemotypes were analyzed through a comparison of full-length transcriptomes and expression profiles. The essential oil of the three *C. porrectum* chemotypes leaves was mainly composed of monoterpenes. In the full-length transcriptome of *C. porrectum*, 104,062 transcripts with 306,337,921 total bp, an average length of 2944 bp, and an N50 length of 5449 bp, were obtained and 94025 transcripts were annotated. In the eucalyptol and linalool chemotype, the camphor and eucalyptol chemotype, and the camphor and linalool chemotype comparison groups, 21, 22 and 18 terpene synthase (TPS) unigenes were identified respectively. Three monoterpene synthase genes, *CpTPS3, CpTPS5 and CpTPS9,* were upregulated in the eucalyptol chemotype compared to the linalool chemotype and camphor chemotype. *CpTPS1* was upregulated in the camphor chemotype compared to the linalool chemotype and the eucalyptol chemotype. *CpTPS4* was upregulated in the linalool chemotype compared to the camphor chemotype and the eucalyptol chemotype. Different unigenes had different expression levels among the three chemotypes, but the unigene expression levels of the 2-C-methyl-D-erythritol 4phosphate (MEP) pathway were generally higher than those of the mevalonate acid (MVA) pathway. Quantitative reverse transcription PCR(qRT-PCR) further validated these expression levels. The present study provides new clues for the functional exploration of the terpenoid synthesis mechanism and key genes in different chemotypes of *C. porrectum*.

## 1. Introduction

*Cinnamomum porrectum* (Roxb.) Kosterm (*C. porrectum*), an evergreen tree of the family Lauraceae and genus Cinnamomum, that grows in evergreen broad-leaved forests or as shrubs, is mainly found in southern China, Bhutan, Cambodia, India, Indonesia, Laos, Malaysia, Myanmar, Nepal, Pakistan, Thailand and Vietnam [1]. The leaves of *C. porrectum* are rich in essential oils, and the whole plant serves as an important source of natural essential oils. *C. porrectum* is widely applied in foods, medicines and the fine chemical industry, including in natural spices and chemical materials [2]. The extracts of the leaves, bark, wood and roots of *C. porrectum* have antioxidant and antibacterial effects [3,4,5], and *C. porrectum* leaves are traditionally used in carminative tonic, stomachic tonic, and antipyretic preparations [6].

Studies have shown that the essential oil of *C. porrectum* leaves is rich in monoterpenes, sesquiterpenes and aromatic compounds [2,7], and the terpenoids with high contents in this leaf essential oil are mainly linalool, eucalyptol, camphor, citral, and nerolidol. According to the first principal component of its leaf essential oil, *C. porrectum* can be divided into the linalool, camphor, eucalyptol, citral and nerolidol chemotypes [2].

Terpenoids in the essential oil of *C. porrectum* are widely used and have a high development value. Among these terpenoids, linalool is frequently used as an odorant for perfumes and in common chemical products; in addition, linalool has analgesic [8], anti-inflammatory [9], and antitumor [10,11] effects. Eucalyptol, with its antimicrobial and insecticidal effects is widely used in medicine, spices and industry. Citral has a strong lemon odor and is widely used in many perfume compositions; in addition, citral has bacteriostatic effects [12].

In recent years, the biosynthesis and metabolic regulation of plant terpenoids have been studied [13,14,15], showing that two independent pathways in plants are involved in the biosynthesis of the 5-carbon structural unit isopentenyl pyrophosphate (IPP) and dimethylallyl diphosphate (DMAPP) of the universal precursor to isomers of terpenoids. In the 2-C-methyl-D-erythritol 4-phosphate (MEP) pathway, the biosynthesis of IPP/DMAPP in plastids begins with pyruvate and glyceraldehyde-3-phosphate [16,17], but in the cytoplasm, the mevalonate (MVA) pathway produces IPP [18,19] from acetyl coenzyme A (CoA). The balance between IPP and DMAPP is controlled by IPP delta-isomerase (IDI), which converts IPP to DMAPP reversibly [20]. Terpenoids are generally formed by catalysis of corresponding precursors by terpene synthases (TPSs), while some terpenoids in plants required further modifications, mainly involving methyltransferases, glycosyltransferases, cytochrome P450 monooxygenase, dehydrogenase and reductase. The existence of these modification enzymes greatly enriches the variety of terpenoids and makes the structure and biological activity of terpenoids tend to be diversified [21]. Monoterpenes were important components of leaf essential oil in *C. porrectum*, and the study of monoterpenes biosynthesis was of great significance. Monoterpene synthase was the key enzyme in monoterpene biosynthesis, and now many monoterpene synthases have been isolated from plants [22,23,24,25,26,27,28,29]. Linalool synthase (LIS) gene was isolated from different source of *Cinnamomum osmophloeum* [26]. The recombinant *LIS* protein can produce s-(+)-linalool from GPP and (E) - Neroli from FPP. Bornyl diphosphate synthase (*LaBPPS*) was cloned from *Lavandula angustifolia* [27], which can catalyze the production of Bornyl or camphor. Through bioinformatics analysis, a monoterpene synthetase was identified from *Laurus nobilis*, and mainly catalyzes the formation of 1,8-cineole [28]. A monoterpene synthetase was cloned from *Cinnamomum tenuipilum*, which was specifically expressed in the geraniol chemotype. After prokaryotic expression and purification, geraniol synthase (*CtGES*) transformed the geraniol diphosphate into a single product geraniol [29]. The genetic transcriptome related to the biosynthesis of terpenoids in *Cinnamomum camphora* was analyzed preliminarily, and some candidate genes involved in the biosynthesis of terpenoids were screened. Three synthetic genes of monoterpene were found to be upregulated in the borneol chemotype [13]. The study of terpenoid synthases and their functions have made great progress. Determining how to use genetic engineering technology to regulate and even transform and improve the composition and yield of the essential oils of plants has become a research hotspot. However, the current research on the terpenoid biosynthetic pathway of *C. porrectum* is still lacking, and there has been no gene study on the related biosynthesis of major terpenoids in the essential oil of *C. porrectum*, directly restricting the genetic improvement and exploitation and utilization of the quality of *C. porrectum* essential oil.

Transcriptome studies provide a useful perspective for expounding the molecular mechanisms of gene functions, cellular reactions, and different biological processes [30]. High-throughput sequencing (HTS) has recently been used to obtain transcriptome data for nonmodel species and provide valuable genomic information, especially for species without genomic sequences [31]. Second-generation sequencing (e.g., Illumina sequencing) has high-throughput capabilities, provides high-quality readings and enables very accurate research on gene expression and quantification. However, existing transcriptome analyses are based on incomplete genomic sequences and incomplete gene annotations. The fragmentation of genes assembled from second-generation sequencing data (usually 100–150 base pairs [bp] in length), which are limited by read length, results in a lack of information on full-length transcripts. With the advantage of an average read length of 10–15 kb, in combination with multifragment library screening technology, single-molecule real-time sequencing, also known as third-generation sequencing (e.g., PacBio sequencing), achieves transcript analysis without the need for splicing, overcomes the limitations of unigenes assembled from short spliced sequences and incomplete transcript structures in traditional second-generation transcriptomes, and can directly obtain all high-quality transcriptome information of single-RNA molecules from the 5′ end to the 3′ end. Third-generation sequencing is applicable to species without genomic sequences and has been used in extensive applications [32,33,34,35,36].

In view of this, the present study carried out third-generation de novo transcriptome sequencing of *C. porrectum* leaf tissue for the first time and sequenced the expression profiles of leaf tissues from three different chemotypes (linalool chemotype, eucalyptol chemotype and camphor chemotype) of *C. porrectum* to reveal information on the transcriptome features of *C. porrectum*. Using kyoto encyclopedia of genes and genomes (KEGG) pathway analysis technology, we explored the genes of *C. porrectum* involved in the metabolism pathways of terpenoids and further analyzed the differentially expressed genes (DEGs) of monoterpenoids in the three different chemotypes. We also preliminarily screened candidate genes for the biosynthesis of several important monoterpenoids. qRT-PCR verification of candidate genes was performed, and key genes in the synthesis of monoterpenoids, such as linalool, eucalyptol and camphor, were screened. This study lays a foundation for the cloning of key genes in the synthesis of important monoterpenoids of *C. porrectum*, functional research, and the genetic improvement and study of the special economic values of *C. porrectum*.

## 2. Results

### 2.1. The Composition of Leaf Extracts from Different Chemotypes of C. porrectum

The leaf essential oil of three chemotypes (the eucalyptol chemotype, Y_L1, Y_L2 and Y_L3; the linalool chemotype, F_L1, F_L2 and F_L3; the camphor chemotype, N_L1, N_L2 and N_L3) of *C. porrectum* were extracted by steam distillation, and the chemical constituents of the leaf essential oil were analyzed by GC/MS. The oil yields of the eucalyptol chemotype, linalool chemotype and camphor chemotype were 11.5–16.5 mg/g, 18.6–21.4 mg/g and 6.1–9.6 mg/g, respectively. A total of 32 compounds were detected in the leaf essential oil, with 20 monoterpenoids, 10 sesquiterpenoids and 2 phenolic compounds. The total content of monoterpenoids accounted for more than 88.04% of the total content of essential oils. In the three chemotypes, the content of the first-principal-component compound was much higher than that of other compounds. The eucalyptol content accounted for more than 34.48% of total content in the eucalyptol chemotype, the linalool content accounted for more than 77.58% of the total content in the linalool chemotype, and the camphor content accounted for more than 92.47% of the total content in the camphor chemotype, that is, the monoterpenoids were almost pure camphor (Table 1).

### 2.2. RNA Sequencing and Transcriptomic Assembly

**Figure 1:**
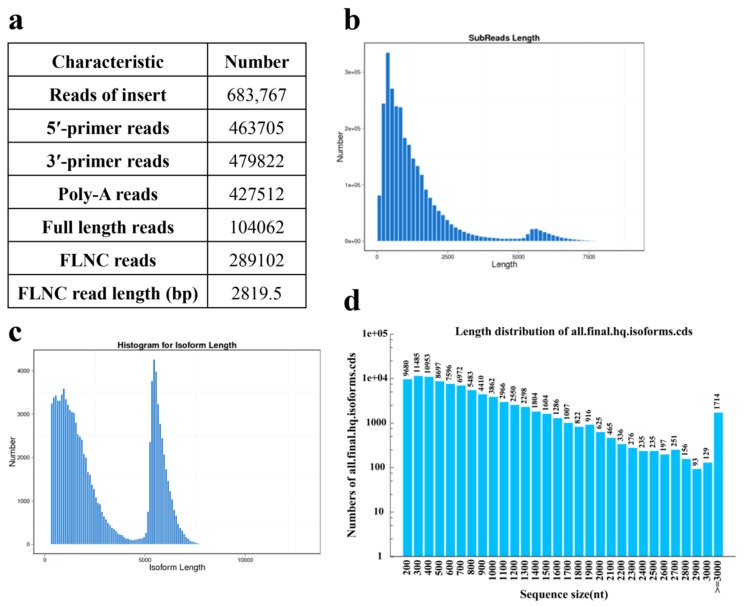
PacBio single-molecule long-read sequencing of *C. porrectum*. (**a**) Reads classification from PacBio Iso-Seq. (**b**) distribution of subreads length. (**c**) Read length distribution. (**d**) Length distribution of all isoforms coding sequence(CDS).

Full-length transcriptome of *C*. *porrectum* was generated from normalized full-length cDNAs from pooled poly(A) RNA of one sample (leaf) and subjected to single molecule real-time (SMRT) sequencing via the PacBio Sequel system. In total, two SMRT cells generated 9,624,949 subreads (13.3 Gb); the average read length was 1389.96 bp and an N50 were 2147 bp. A total of 683,767 reads of insert (ROIs) were generated and 104,062 those sequences containing two primers and a poly-A tail were identified as full-length reads of inserts(ROIs), which were subsequently classified into chimeric and nonchimeric reads. Of these ROIs, 289,102 were identified as full-length non-chimeric (FLNC) reads with an average read length 2819.5 bp (Figure 1a). Full-length nonchimeric sequences were clustered, and with the Quiver algorithm, each cluster was corrected and integrated into a consensus sequence. The consensus sequence output by Quiver was divided into low-quality sequences (low QV, LQ) and high-quality sequences (high QV, HQ). A total of 213586 full-length consensus transcripts, including 133,966 HQ (> 99% accuracy) transcripts and 79620 LQ transcripts, were generated. Information on each library of clustered reads is shown in Table S1, and the sequence length and sequence quality of the low-QV and high-QV sequences were compared (Figure S1). Subsequent analyses only used high-QV consensus sequences. Iso-Seq produced more unigenes with lengths > 2000 bp than Illumina sequencing.

These results indicated that PacBio Iso-Seq provides a practical method for producing full-length transcripts without assembly steps and is an important improvement for transcriptome studies of species without reference genomes.

Library clusters and high-quality sequences obtained after error correction were finally merged to eliminate redundancy. After this process, the transcriptome of *C. porrectum* had a total of 104,062 transcripts with a total of 306,337,921 bp; the average sequence length was 2944 bp, and the N50 length was 5449 bp (Table 2). For the length distribution of subreads, see Figure 1b, and the length distribution of nonredundant transcripts is shown in Figure 1c.

After clustering and error correction, the TransDecoder software was used to identify the candidate coding regions in each transcript. First, the longest open reading frame was extracted. Then, using BLAST, the SwissProt database was compared with Hmmscan to search for homology to Pfam domains to predict the coding regions. A total of 89,103 complete transcripts were obtained. The details of the prediction results are listed in Table 3, and the coding sequence(CDS) length distribution is shown in Figure 1d.

### 2.3. Gene Annotation and Functional Classification

**Figure 2:**
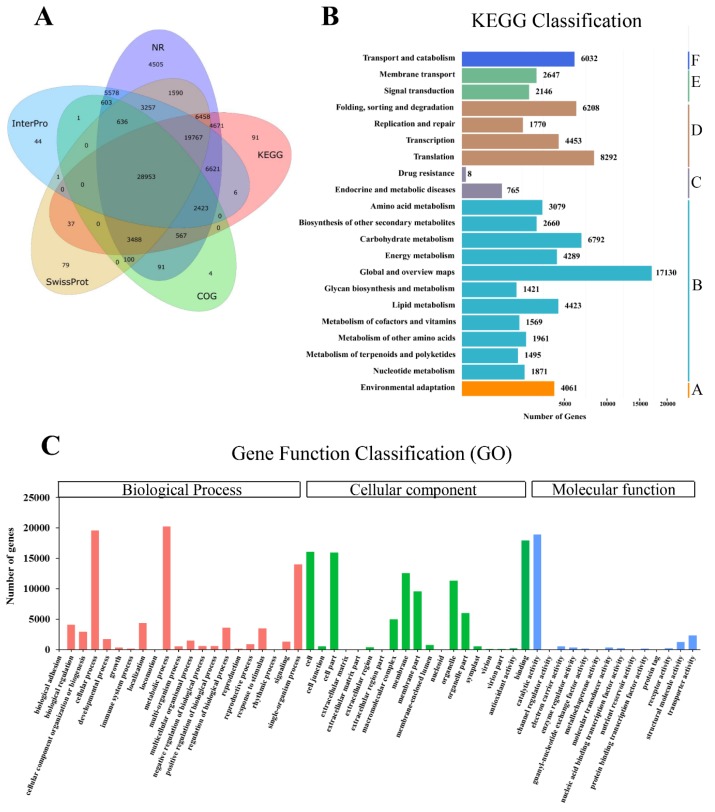
Functional annotation and classification of assembled unigenes in *C. porrectum*. (**A**) Annotation information of the Venn diagram. (**B**) KEGG pathway classification of putative proteins. The unigenes were divided into six branches according to the following KEGG metabolic pathways: organismal systems A. metabolism B. human diseases C. genetic information processing D. environmental information processing E. and cellular processes F. (**C**) GO classification of unigenes in *C. porrectum.* GO terms are classified into three main categories: biological process (BP), cellular component (CC) and molecular function (MF).

After transcripts were clustered and corrected, functional annotations (NCBI non-redundant protein sequences(NR), NCBI non-redundant nucleotide sequences(NT), Gene ontology(GO), EuKaryotic Orthologous Groups(KOG), KEGG, SwissProt, Interpro) were performed using BLAST, Blast2GO and InterProScan5, and a total of 104,062 transcripts were annotated (Figure 2A). The total number of transcripts annotated by any database was 94,025, accounting for 90.53% of all transcripts. The NR database had the highest annotation rate, accounting for 85.52% of all transcripts and the GO database had the lowest annotation rate, accounting for 34.26% of all transcripts. The total number of transcripts annotated by all seven databases was 21,781, accounting for 20.93% of all transcripts. The overall status of the annotations is shown in Table S2. To better understand the full-length transcripts that were obtained, their functions were investigated using the KEGG pathway database; a total of 104,062 genes were involved in 19 metabolic pathways. The unigenes were divided into six branches according to the KEGG metabolic pathway: organismal systems (A), metabolism (B), human diseases (C), genetic information processing (D), environmental information processing (E), and cellular processes (F). It was found that 1495 unigenes were enriched in the metabolic pathways of terpenoids and polyketides (Figure 2B). The obtained unigenes were GO annotated, and the genes that were annotated successfully were classified according to the 54 biological functional classifications under the three major GO categories: biological process, cellular component and molecular function. In the biological process category, there were relatively high numbers of genes involved in the cellular process, metabolic process, and single-organism process classifications. In the cellular component category, there were more genes involved in the cell, cell part, membrane, and binding processes classifications than in other classifications. In the molecular function category, genes were mainly classified into catalytic activity, structural molecule activity and transporter activity (Figure 2C). The above results reveal a comprehensive functional characterization of the full-length *C. porrectum* transcriptome, and will help further study on gene functions.

### 2.4. The Identification of Relative DEGs in C. porrectum Chemotypes and Enrichment Analysis of Transcripts

The expression profiles of the *C. porrectum* leaf tissues from the eucalyptol, linalool and camphor chemotypes underwent sequencing analysis. The flux and quality of the RNA-Seq data are shown in Table 4. There was a total of 384.31 M clean read data obtained from the three different chemotypes with three biological repetitions, and the total degree of mapping was between 73% and 82%.

**Figure 3:**
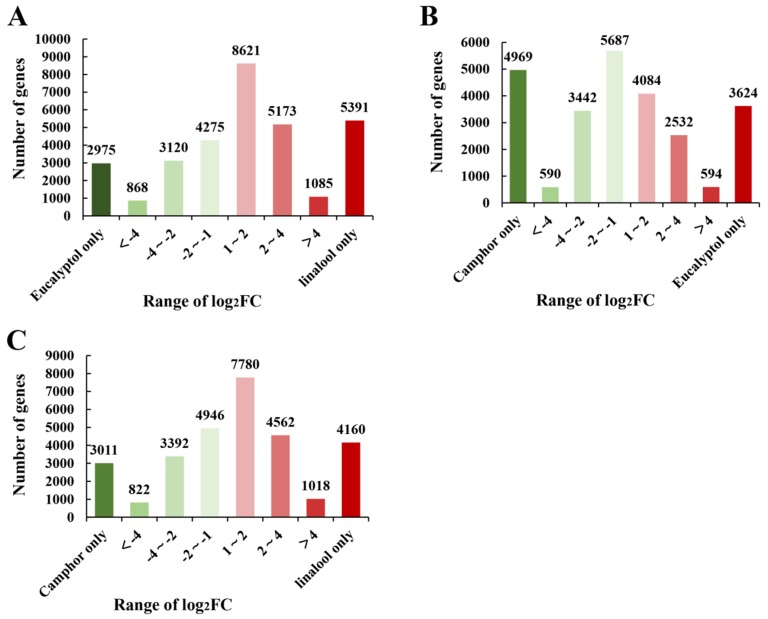
(**A**) Number of DEGs in the eucalyptol chemotype and linalool chemotype of *C. porrectum*. Red: upregulated genes; green: downregulated genes. Relative expression of DEGs selected at a *Q*-value < 0.05. The fold change (FC) was calculated as the ratio between the linalool chemotype and eucalyptol chemotype. The x-axis represents the range of Log2 FC. The y-axis indicates the number of detected DEGs. (**B**) Number of DEGs in camphor chemotype and eucalyptol chemotype of *C. porrectum*. The fold change (FC) was calculated as the ratio between the eucalyptol chemotype and camphor chemotype. (**C**) Number of and DEGs in camphor chemotype and linalool chemotype of *C. porrectum.* The fold change (FC) was calculated as the ratio between the linalool chemotype and camphor chemotype.

To fully explore the potential differential gene expression between the linalool and eucalyptol chemotypes, between the camphor and linalool chemotypes, and between the camphor and eucalyptol chemotypes, the clean reads were mapped to the unigene database. Gene expression data were standardized using a variety of calibration methods, and the DEGs between different chemical types were characterized by differentially expressed sequence (DESeq) with a *Q*-value < 0.05 and |log2. Fold_change| > 1 [41]. In the eucalyptol chemotype and linalool chemotype comparison group, a total of 31,508 unigenes were identified, including 20,270 upregulated genes and 11,238 downregulated genes. A total of 2975 unigenes were unique to the eucalyptol chemotype, 5391 unigenes were unique to the linalool chemotype, and 23,142 unigenes were shared by the two chemotypes (Figure 3A). In the camphor chemotype and eucalyptol chemotype comparison group, a total of 25,522 unigenes were identified, including 10,834 upregulated genes and 14688 downregulated genes. Of these unigenes, 4969 were unique to the camphor chemotype, 3624 were unique to the eucalyptol chemotype, and 16,929 were shared by the two chemotypes (Figure 3B). In the camphor chemotype and linalool chemotype comparison group, a total of 29,691 unigenes were identified, including 17520 upregulated genes and 12,171 downregulated genes. A total of 3011 unigenes were unique to the camphor chemotype, 4160 unigenes were unique to the linalool chemotype, and 22,520 genes were shared by the two chemotypes (Figure 3C). To explore the differences in metabolic pathways between the linalool and eucalyptol chemotypes, between the camphor and linalool chemotypes, and between the camphor and eucalyptol chemotypes, KEGG enrichment pathways were analyzed using 31,508, 25,522 and 29,691 DEGs, respectively. The top 20 KEGG pathway categories are presented in the form of a dot plot (Figure S2A–C). The pathway with the most significant enrichment of DEGs in the camphor and linalool chemotype comparison group was the “monoterpene biosynthesis” pathway.

The “monoterpene biosynthesis” pathway was also enriched significantly in the camphor and eucalyptol chemotype comparison group.

### 2.5. Candidate Genes Involved in Terpenoid Biosynthesis

**Figure 4:**
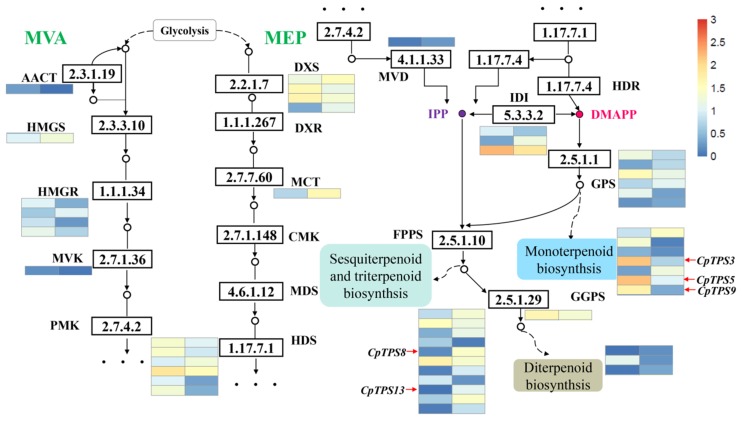
Differentially expressed genes (DEGs) in terpenoid backbone biosynthesis in the eucalyptol chemotype and linalool chemotype comparison group of *C. porrectum*. Enzymes expression patterns are indicated with the value of log (FPKM+ 1). The expression pattern is shown within two columns, with the left column representing the eucalyptol chemotype and the right representing the linalool chemotype. Acetyl-CoA C-acetyltransferase (CCAT); Hydroxymethylglutaryl-CoA synthase (HMGS); Hydroxymethylglutaryl-CoA reductase(HMGR); Mevalonate kinase (MVK); Phosphomevalonate kinase (PMK); Diphosphomevalonate decarboxylase (MVD); 1-deoxy-D-xylulose-5-phosphate synthase (DXS); 1-deoxy-Dxylulose-5-phosphate reductoisomerase (DXR); 2-C-methyl-D-erythritol 4-phosphate cytidylyltransferase (MCT); 4-diphosphocytidyl-2-C-methyl-D-erythritol kinase (CMK); 2-C-methyl-D-erythritol 2,4-cyclodiphosphate synthase (MDS); (E)-4-hydroxy-3-methylbut-2-enyl- diphosphate synthase (HDS); 4-hydroxy-3-methylbut-2-en-1-yl diphosphate reductase(HDR); isopentenyl-diphosphate Delta-isomerase (IDI).

To explore the regulatory mechanisms for the accumulation patterns of different terpenoids in *C. porrectum*, the expression profiles of genes involved in terpenoid biosynthesis were analyzed. In the eucalyptol chemotype and linalool chemotype, the camphor chemotype and eucalyptol chemotype, and the camphor chemotype and linalool chemotype comparison groups, 52, 49 and 66 DEGs for the biosynthesis of terpenes were identified, respectively (Figure 4, Figure S3 and Figure S4). The expression level of all these unigenes are shown in the Figure 4, Figures S3 and S4, and the fragments per kilobase per million mapped (FPKM) values are shown in Table S3. Most of the genes exhibited a high transcriptome expression level, which encoding key enzymes in the MEP and MVA pathway (KEGG entry ko00900). The expression levels of different unigenes among the three different chemotypes were different, but the unigene expression level of the MEP pathway was higher than that of the MVA pathway as a whole (Figure 4, Figures S3 and S4). Both MEP and MVA pathways produce IPP and its isomer, dmapp. The active biosynthesis of building blocks contributes to the accumulation of various terpenoids, which is consistent with our analysis in the components of the extracts from the leaves of *C. porrectum (*Table 1). Moreover, in the eucalyptol chemotype and linalool chemotype comparison group, we screened 21 unigenes encoding TPS, namely, 7 monoterpene synthases, 3 diterpenoid synthases and 11 sesquiterpene and triterpene synthases (Figure 4). The eucalyptol chemotype upregulated 5 unigenes of monoterpene synthases, 1 unigene of diterpene synthase and 4 unigenes involving sesquiterpene and triterpene synthases. However, in the linalool chemotype, 2 unigenes of monoterpene synthases, 2 unigenes of diterpene biosynthesis, and 7 unigenes of sesquiterpene and triterpene synthases were upregulated (Figure 4). In the camphor chemotype and eucalyptol chemotype comparison group, we screened 22 unigenes encoding TPS, namely, 9 monoterpene synthases, 3 diterpenoid synthases and 10 sesquiterpene and triterpene synthases (Figure S3). The camphor chemotype upregulated 3 unigenes of monoterpene synthases, 2 unigenes of diterpene synthases and 6 unigenes of sesquiterpene and triterpene synthases. However, in the eucalyptol chemotype, 6 unigenes of monoterpene synthases, 1 unigene of diterpene biosynthesis, and 4 unigenes of sesquiterpene and triterpene synthases were upregulated (Figure S3). In the camphor chemotype and linalool chemotype comparison group, we screened 18 unigenes encoding TPS, namely, 5 monoterpene synthases, 2 diterpenoid synthases and 11 sesquiterpene and triterpene synthases (Figure S4). The camphor chemotype upregulated 2 unigenes of monoterpene synthases, 2 unigenes of diterpene synthase and 5 unigenes of sesquiterpene and triterpene synthases. However, in the linalool chemotype, 3 unigenes of monoterpene synthases and 6 unigenes of sesquiterpene and triterpene synthases were upregulated, and all the unigenes of diterpene synthases were downregulated (Figure S4). On the whole, the observed high expression levels of enzymes in the terpene skeleton pathway were consistent with the high rate of terpenoid synthesis and suggest that the different terpenoid compositions in the three chemotypes may arise from differences in the expression levels of TPS genes.

### 2.6. Construction of Phylogenetic Tree of TPS

**Figure 5:**
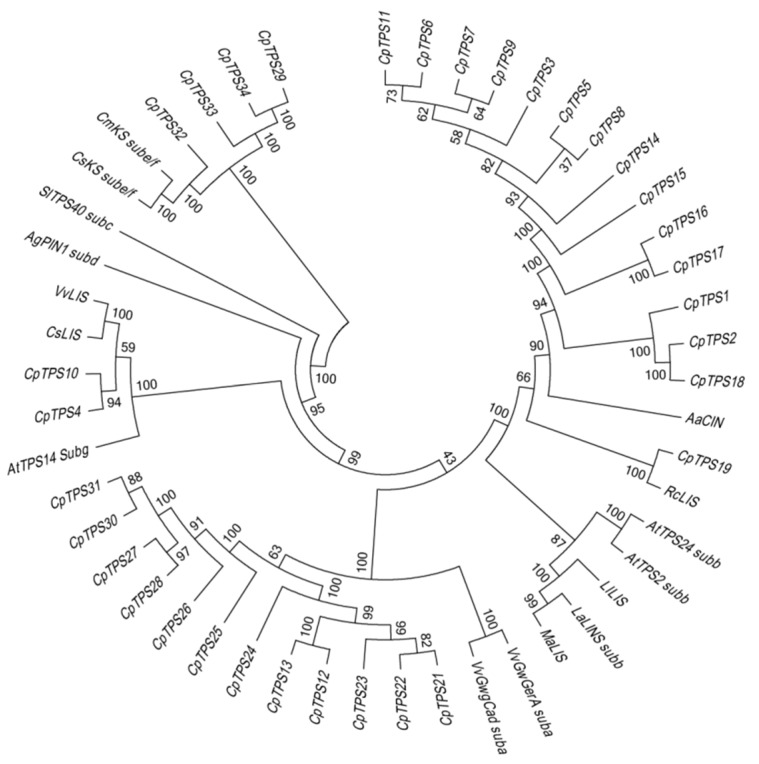
Results of phylogenetic tree of *CpTPS* gene family and representative terpene synthase of the known function in other plants using the neighbor-joining method by MEGA7 software. Bootstrap values are shown as a percentage of 1000 replicates. The sequences of genes in phylogenetic tree of *CpTPS* were shown in Table S4. The accession number for characterized TPS in the phylogenetic tree are: *AtTPS2*(NP 193406.3); *AtTPS14*(NP 001185286.1); *AtTPS24*(NP 189209.2); *SlTPS40* (NP 001234008.2); *AgPIN1* (O24475.1); *VvGwGerA* (ADR66821.1); *VvGwgCad* (ADR74199.1); *LaLINS* (Q2XSC5.1); *CmKS*(XP_022968895.1); *CsKS*(NP_001292675.1); *VvLIS*(ADR74212.1); *LiLIS*(ABD77417.1); *RcLIS*(AVR48793.1); *MaLIS*(AAL99381.1); *AaCIN*(PWA51422.1)*; CsLIS*(AGX26045.1). The function of known TPS genes in the phylogenetic tree were shown in Table S8.

Terpene synthase is the main cause of the diversity of terpenoids. The mechanism of terpenoid accumulation in different chemotypes of *C. porrectum* was investigated by constructing a phylogenetic tree of TPS (Figure 5). In combination with terpene synthase genes with currently known functions, we constructed a phylogenetic tree of TPS sequences and divided the terpene genes into six subfamilies. Among these subfamilies, the b subfamily responsible for the synthesis of monoterpenoids had the largest number of genes, with 15 members, while there were 12 genes in a subfamily responsible for the synthesis of sesquiterpenes, 0 genes in the c subfamily, 0 genes in the d subfamily, 4 genes in the e/f subfamily, and 2 genes in the g subfamily.

### 2.7. qRT-PCR Validation of DEGs from the RNA-Seq Analysis

**Figure 6:**
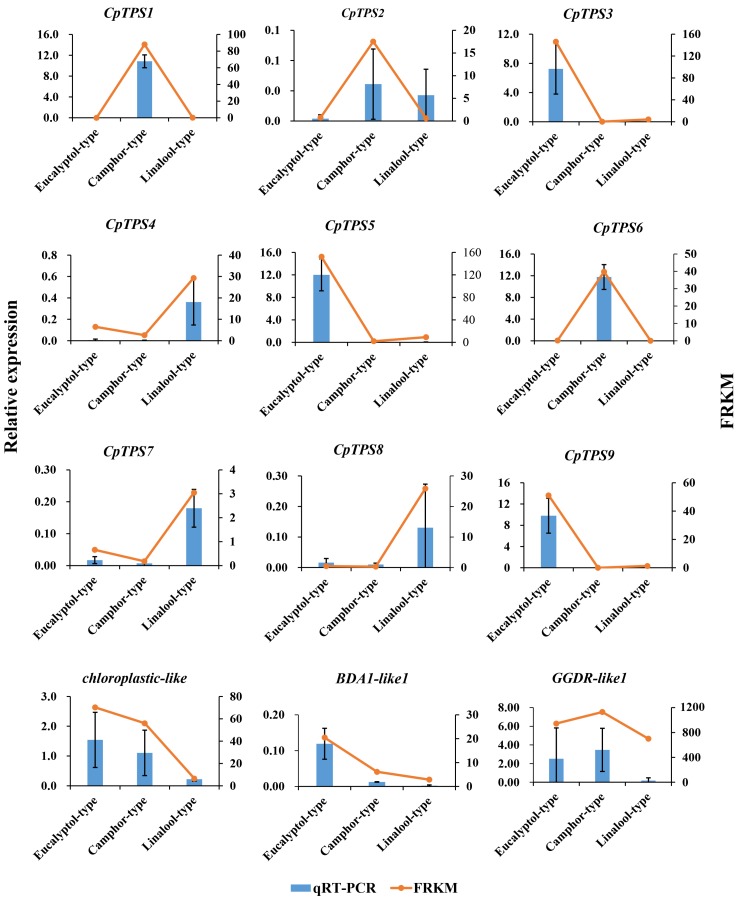
Expression analyses of selected genes (Table S5) using both qRT-PCR and RNA-Seq (FC of FPKM). The relative expression levels were estimated from the threshold of the PCR cycle with the delta CT method. The error bars indicate the standard errors of three biological replicates. Composition of the leaf extracts of the three chemotypes are shown in Table 1.

**Figure 7:**
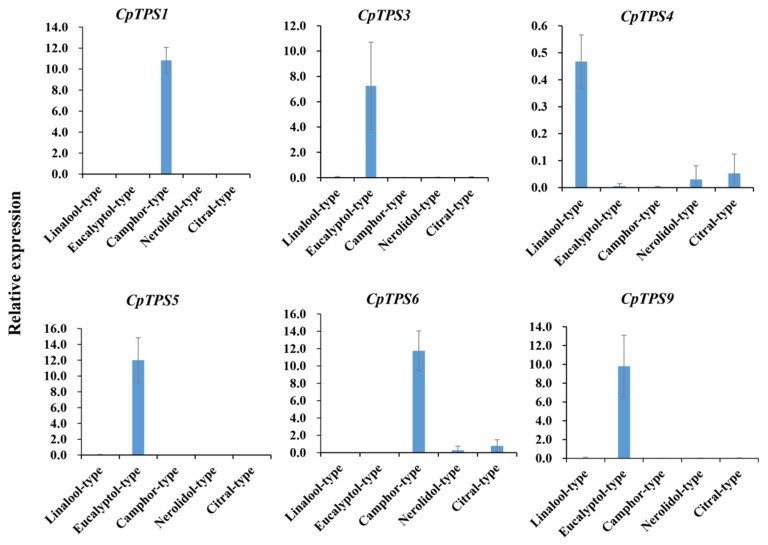
qRT-PCR validation of selected genes in five chemotypes of C. porrectum. The five chemotypes are the linalool-, eucalyptol-, camphor-, nerolidol and citral-type. Relative expression levels were estimated from the threshold of the PCR cycle using the Delta CT method. The error bars indicate the standard errors of three biological replicates. Composition of the leaf extracts of the linalool-type, eucalyptol-type and camphor-type are shown in Table 1; Composition of the leaf extracts of nerolidol-type and citral-type are shown in Table S9.

To verify the expression pattern of the terpene biosynthetic genes obtained from RNA-Seq analysis, the expression of 12 unigenes was examined by qRT-PCR (Figure 6). The qRT-PCR-determined expression levels of these genes were generally consistent with the expression levels deduced from RNA-Seq FPKM data (Figure 6). The results confirm the reliability of the transcriptomic profiling data from RNA-Seq. In addition, we selected 6 of these DEGs and detected their expression in five chemotypes (linalool-, eucalyptol-, camphor-, nerolidol- and citral-type) using qRT-PCR. Notably, *CpTPS3*, *CpTPS5* and *CpTPS9* were specifically expressed in the eucalyptol chemotype; *CpTPS1* was specifically expressed in the camphor chemotype; *CpTPS4* was expressed in all five chemotypes, with the maximum expression in the linalool chemotype. These results, which basically agree with the transcriptome results, also validate the reliability of the transcriptome (Figure 7).

## 3. Discussion

At present, research on the *Cinnamomum camphora* transcriptome has mainly been based on cost-effective next-generation RNA sequencing [13]. However, because of the short read length of this method, it is difficult to obtain an accurate full-length sequence using next-generation RNA sequencing without a reference genome [42]. PacBio Iso-Seq, the most popular application of third-generation sequencing, has proven useful for accurately characterizing the diverse landscape of isoforms, as this method can obtain full-length transcripts [43]. Using the PacBio Iso-Seq method, this study provided the first comprehensive set of full-length isoforms in *C. porrectum*. After eliminating redundancy, a total of 104,062 transcripts were obtained, with a total of 306,337,921 bp, an average sequence length of 2944 bp, and an N50 length of 5449 bp. Based on these highly accurate transcripts, a total of 104,062 transcripts were successfully annotated by at least one of seven known functional protein databases (NR, NT, GO, KOG, KEGG, SwissProt, Interpro). Furthermore, 104,062 genes were involved in a metabolic pathway of 19 KEGG pathway databases, and 1495 unigenes were found to be enriched in pathways of terpenoid and polyketide metabolism. These results indicate that PacBio transcriptome sequencing has a high ability to generate full-length transcript sequence information, providing an important reference for genome annotation and gene function analysis.

In recent years, genes associated with terpene biosynthesis have been studied extensively in different plants. Lauraceous plants are rich in terpenoids, and several genes involved in the biosynthesis of terpenoids have been successfully identified, and their functions have been explored [26,29]. Three TPS genes encoding monoterpene synthases were isolated in *Litsea cubeba*: *LcTPS1*, which converts geranyl diphosphate to trans-ocimene, *LcTPS2*, which converts geranyl diphosphate to α-thujene, and *LcTPS3*, a mutifunctional enzyme that converts geranyl diphosphate to α-thujene and (+)- sabinene [44]. In *Laurus nobilis*, the formation of 1,8-cineole, cadinenes and geranyllinalool catalyzed by TPS enzymes was characterized [28]. Sixty-seven unigenes were isolated from the transcriptome of *C. camphora,* which might be involved in terpene biosynthesis. The expression of *TPS14-like1, TPS14-like2* and *TPS14-like3* were upregulated in the borneol chemotype relative to the linalool chemotype. These results are helpful to better understand the differential accumulation of terpenoids in different chemotypes of C. camphora [13]. This study analyzed the transcriptomes of three chemotypes of *C. porroctum*. In the eucalyptol chemotype and linalool chemotype, the camphor chemotype and eucalyptol chemotype, and the camphor chemotype and linalool chemotype comparison groups, 52, 49, and 66 candidate unigenes, respectively, were identified to be related to terpene biosynthesis. Furthermore, the terpenoid synthase genes *CpTPS1, CpTPS3, CpTPS4, CpTPS5* and *CpTPS9* had specific expression or higher expression in one of the chemotypes and may be involved in monoterpene biosynthesis.

It is now generally accepted that MVA and MEP pathway activities are regulated at both the transcript and protein levels to control precursor availability [45]. Gene coexpression networks have revealed that there is no en bloc transcriptional regulation of all genes encoding MVA and MEP pathway enzymes. Isoprenoids synthesized via MVA and MEP pathways are controlled by independent regulatory networks with restricted connectivity [45].

HMGR and DXS are two rate-limiting enzymes in the MVA pathway [46] and the MEP pathway [47]. In the transcriptomes of the linalool- and borneol-chemotypes of *C. camphora*, 30 unigenes, which were highly homologous to 14 known enzymes, were annotated in the MVA and MEP pathways of terpene skeleton biosynthesis in the borneol, and high transcriptome expression levels were shown in both chemotypes [13]. In of *C. porrectum*, there were 21 unigenes encoding TPS in the eucalyptol chemotype and linalool chemotype comparative group, 5 monoterpene synthase unigenes upregulated in the eucalyptol chemotype, and 2 monoterpene synthase unigenes upregulated in the linalool chemotype. There were 22 unigenes encoding TPS in the camphor chemotype and eucalyptol chemotype comparative group, 3 upregulated monoterpene synthase unigenes in the camphor chemotype, and 6 upregulated monoterpene synthase unigenes in the eucalyptol chemotype. There were 18 unigenes encoding TPS in the camphor chemotype and linalool chemotype comparative group, 2 upregulated monoterpene synthase unigenes in the chemotype camphor, and 3 upregulated monoterpene synthase unigenes with upregulated expression in linalool chemotype. These results indicate that there may be differences in the gene expression patterns for the regulation and formation of monoterpenoids among different chemotypes. The MEP pathway provides precursors for the synthesis of monoterpenes and diterpenes in plastids, while sesquiterpenes are derived from precursors of the MVA pathway in the cytoplasm [45]. The unigene expression level of the MEP pathway was higher than that of the MVA pathway indicating the abundance of monoterpenes. Crosstalk between these two different terpene skeleton pathways has been documented, and the relative contribution of each pathway to terpene biosynthesis remains uncertain.

TPS genes is responsible for the synthesis of diverse terpenoids molecules from two isomeric isoprene precursors. The phylogenetic tree of *C. porrectum* with the characterized TPS proteins is divided into seven clades, with a majority expansion of TPS-a and TPS-b clades, indicating gene family expansion is correlated with mono- and sesquiterpene diversity.

Monoterpenes are the principal components of many member leaf extracts of *Cinnamomum* species, such as *Cinnamomum osmophloeum* [26], *Cinnamomum kanehirae* [48] and *Cinnamomum camphora* [13]. In this paper, 33 components were identified in the essential oil from leaf tissue of the eucalyptol chemotype, linalool chemotype and camphor chemotype of *C. porrectum*. Analysis of the components showed that the essential oils isolated from the three chemotypes were mainly composed of monoterpenes, including 20 monoterpenoids, basically complying with the study results in *C. camphora* [13]; however, there were significant differences in the varieties and contents of monoterpenoids among the three chemical types. The content of linalool in the linalool chemotype was 77.58%-87.88%, but the linalool content was very low in the other two chemotypes. The content of eucalyptol in the eucalyptol chemotype was 34.48–35.63%, but the eucalyptol content was extremely low in the other two chemotypes. Notably, the monoterpenoid content of the camphor chemotype was almost pure camphor, but camphor was absent or present in a small amount in the other two chemotypes. The difference in the contents of the three main monoterpenoids (linalool, eucalyptol and camphor) in the three chemotypes of *C. porrectum* greatly coincided with the differential expression of monoterpene synthase genes, such as *CpTPS1, CpTPS3, CpTPS4, CpTPS5* and *CpTPS9*, among the three different chemotypes. Presumably, the *CpTPS4* gene is highly likely to affect the synthesis of linalool in *C. porrectum* leaf tissue, the *CpTPS3, CpTPS5* and *CpTPS9* genes affect the synthesis of eucalyptol, and the *CpTPS1,* gene is related to the synthesis of camphor. Compared with the published proteins, *CpTPS4* had the highest similarity with S- +-linalool synthase of *Cinnamomum microratum* (94.44%, Accession RWR97874.1), and *CpTPS1* had the highest similarity with geraniol synthase of *Cinnamomum microratum* (96.55%,Accession RWR98053.1). *CpTPS3*, *CpTPS5* and *CpTPS9* had the highest similarity with alpha thujene synthase/sabinene synthase of *Litsea cubeba* (Accession AEJ91556.1), and the similarities were 90%, 90.21% and 90.69% respectively. Sequence similarity alone can not predict the specific biochemical function of a single TPS family member, because only a few amino acids can cause dramatic changes in the terpenoid structure of a given TPS enzyme [49,50]. In addition, many TPS are prolific enzymes, which can generally produce mixtures of different proportions of the same compounds [51]. In the study of amino acid sequence similarity of limonene synthases in different species, it was also displayed that sequence similarity was more closely related to genetic relationship than function [24,25]. Therefore, it is difficult to judge the specific functions of genes from the similarity of sequences. The specific functions of these five genes need to be further verified. The future functional verification and utilization of these monoterpenoids should focus on these genes.

## 4. Materials and Methods

### 4.1. Plant Materials

Three generations of leaf tissue samples for full-length transcriptome sequencing were collected from the Guangdong Baixi Nature Reserve in southern China in June 2017 (23.7129°N, 115.1971°E, Guangdong, China). Five leaf samples of *C. porrectum* were gathered randomly, with an interval of more than 300 m between sample plants, the sampled trees were approximately 20 years old. In the middle and upper part of the tree crown, a whole leaf was taken from the East, West, North and south respectively for RNA extraction. RNA was extracted separately, and the five samples of RNA were mixed in equal amounts for transcriptome sequencing. The leaf samples with linalool, eucalyptol or camphor as the first principal components of essential oil were collected separately and quickly frozen in liquid nitrogen. Leaf samples were placed in a refrigerator at −80 °C for the sequencing of different chemotypes of leaf tissue, and there were three biological replicates for each chemotype. At the same time, sampling was performed separately to determine the content of the essential oil and the chemical composition of the essential oil.

### 4.2. Determination of Chemical Composition

Isolation of essential oil: Samples of fresh *C. porrectum* leaves (about 200 g) were each weighed immediately after being picked and were kept under hermetic conditions at a low temperature. The volatile essential oil was isolated by steam distillation with a distillation time of 2 h. Isolation of essential oil from each sample was repeated three times. After the essential oil was weighed, it was stored under hermetic conditions at low temperature.

Gas chromatography-mass spectrometry: GC/MS: GC/MS analysis was performed using a Shimadzu QP2020 GC/MS instrument (chromatographic column: SH-RXI-5SILMS, 30 m × 0.25 mm × 0.25 µm). GC/MS procedure: kept at 80 °C for 2 min, elevated to 160 °C at 8 °C/min, and then raised to 250 °C at 8 °C/min, maintained for 2 min. The injection volume was 1.0 µL, and a split ratio of 20:1 was used. The temperature of the injection port was 280 °C, the EI ion source was 230 °C, and the connection line was 200 °C. The MS scan range (*m*/*z*) was 50–650. For analysis, essential oils were dissolved in alcohol (30 mg/ mL) and directly injected.

Component identification: The components were identified by gas chromatography. The retention index of n-alkanes (c9-c33) was determined under the same operating conditions by comparison with the retention index of literature or the national institute of standards and technology (NIST) 8.0 standard. The retention index of n-alkanes (c9-c33) was further determined by comparison with the retention index of literature or NIST 8.0 standard. Their mass spectra of two columns were compared with those stored in NIST 8.0 libraries or with mass spectra from the available literature [38,39,40] to further identify the components.

Quantitative analysis: Calibration curve of each of the standard compounds, β-pinene, eucalyptol, 2-Dodecanone and eugenol was generated using six concentration points. The response factor of the respective standard and the percentage of each chemical constituent in the essential oil were determined by following the reported procedure [52,53].

### 4.3. RNA Extraction and Library Construction

RNA was extracted using the Huayueyang polysaccharide and polyphenol plant RNA extraction kit. The total RNA concentration, RIN value, 28S/18S and fragment size were detected using the Agilent 2100 Bioanalyzer, and RNA purity detection was conducted with NanoDropTM. Total RNA was first synthesized into first-strand cDNA using the Clontech SMARTer PCR cDNA Synthesis Kit, first-strand cDNA was synthesized into second-strand cDNA through PCR amplification, and then double-stranded DNA was used for SMRTbell library construction after secondary PCR amplification. One library > 4K for fragment selection and one nonfragmented library were constructed with BluePippinTM. The two libraries were mixed for sequencing.

### 4.4. Transcriptome Sequencing and Assembly

Three-generation sequencing of the *C. porrectum* transcriptome library was performed using the PacBio Sequel platform. According to the offline subread data, each inserted fragment was identified and integrated to generate the consensus form of the inserted fragment sequence. The identified inserted fragment sequence was subject to dejointed poly-A sequencing, and then classified as a full-length, non-full-length, chimeric, or nonchimeric sequence. With the ICE algorithm, de novo prediction of isoforms was conducted. The Quiver algorithm was used for isoform correction, and high-QV and low-QV isoforms were output. Redundancy was eliminated from the clustered and error-corrected transcripts using cd-hit to obtain a high-quality full-length unigene library.

### 4.5. Determination of the Expression Profiles of Three Chemotypes

RNA was sequenced on an Illumina HiSeq 2500 platform (Illumina Inc., San Diego, CA, USA) with three biological replicates in this study. Sequencing libraries were generated using the NEBNext^®^ Ultra™ RNA Library Prep Kit for Illumina^®^ (NEB, Boston, MA, USA) following the manufacturer’s recommendations. The number of fragments per kilobase of exon per million fragments mapped (FPKM) of unigenes were calculated using the RSEM (v1.2.15) software to normalize the relative gene expression levels [54,55].

### 4.6. Transcriptome Annotation and Differential Expression Analysis

Leaf transcriptome functional annotation was performed using BLAST program with an E-value cut-off of 1 × 10^−5^ against the following databases: NR, InterPro, SwissProt, COG and KEGG. Gene ontology (GO) was analyzed by Blast2GO version 2.5 [56]. Read counts of different chemotypes of *C. porrectum* samples were imported to the DESeq R package (v1.10.1) for differential analysis [57]. The differential expressed transcripts with false discovery rate (FDR) < 0.05 were exported for downstream analysis.

### 4.7. Construction of Phylogenetic Tree of TPS

Based on the results of the functional annotation, the potential sassafras TPS protein sequence in the transcriptome was obtained and manually corrected for redundancy. First, in the phylogenetic analysis of sassafras TPS, MUSCLE was used to conduct protein sequence alignment to obtain the conserved domain sequence, and then a phylogenetic tree was constructed using MEGA7 with the neighbor-joining (NJ) method and 1000 bootstrap replicates [58].

### 4.8. qRT-PCR Validation of DEGs

qRT-PCR was employed using the SYBR Green PCR Master Mix (Takara, Beijing, China) on the Roche-LightCycler-480. The primers were designed using Oligo 7.0 (Molecular Biology Insights, Cascade, CO, USA). *CpTPS1, CpTPS2, CpTPS3, CpTPS4, CpTPS5, CpTPS6, CpTPS7, CpTPS8, CpTPS9, chloroplastic-like, GGDR-like1 and BDA1-like1* genes were performed. Details of the primers used for the qRT-qPCR assay are listed in Table S5, and sequences of these unigenes are listed in Table S6. The cDNA was synthesized using the PrimeScriptTM RT Reagent Kit (Perfect Real Time) reverse transcription kit, and the actin (*ACT*) gene was used as the internal reference gene. According to the quantitative reagent SYBR^®^ Premix Ex TaqTM II (Tli RNaseH Plus) instructions, all samples were subjected subjected to three biological replications. The Ct values for all genes were normalized to the Ct value of actin [59].

## 5. Conclusions

In this study, the transcriptome and metabolism of leaf tissue from the linalool, eucalyptol and camphor chemotypes of *C. porrectum* were analyzed. These data describe the gene expression levels during terpene biosynthesis in *C. porrectum*. Monoterpenes were identified as the major component of the three chemotypes. The full-length transcriptome had a total of 104,062 transcripts, including 89,103 complete transcripts, and was annotated, laying the foundation for further functional studies. KEGG enrichment analysis found that 1495 unigenes were enriched in the metabolic pathways of terpenoids and polyketides. In the eucalyptol and linalool chemotype, the camphor and eucalyptol chemotype, and the camphor and linalool chemotype comparison groups, 52, 49 and 66 terpene biosynthesis DEGs were identified. GO and KEGG enrichment analysis indicated that TPS activity and oxidoreductase activity could explain the differential accumulation of terpenoids among the three chemotypes of *C. porrectum*, but further functional studies are required to elucidate the regulatory mechanism of the formation of terpenoids. In conclusion, the full-length transcriptome sequence and the gene expression profiles of this study provide valuable information for understanding the accumulation of terpenoids in different chemotypes of *C. porrectum*, lay a foundation for research on the function of key genes in the synthesis of terpenoids, and offer clues for the study of the terpenoid formation mechanism in *C. porrectum*.

## Figures and Tables

**Table 1:** Composition of the leaf extracts of C. *porrectum.*

RIa	RIb	Component	Y_L1 (%)	Y_L2 (%)	Y_L3 (%)	F_L1 (%)	F_L2 (%)	F_L3 (%)	N_L1 (%)	N_L2 (%)	N_L3 (%)
Monoterpenes			92.98	93.65	90.04	89.46	88.04	88.33	99.01	92.47	98.32
929	928	3-Thujene	0.51	0.51	0.28	-	-	-	-	-	-
939	935	*α*-Pinene	3.16	3.47	2	-	-	-	-	-	-
977	1006	*β*-Phellandrene	12.16	9.9	9.53	-	-	-	-	-	-
986	978	*β*-Pinene	3.47	3.41	2.55	-	-	-	-	-	-
989	988	Myrcene	1.78	1.99	1.76	-	-	-	-	-	-
1011	1002	*α*-Phellandrene	0.37	0.46	0.16	-	-	-	-	-	-
1021	1014	*α*-Terpinene	1.5	2.39	1.57	-	-	-	-	-	-
1033	1031	D-Limonene	-	-	-	tr	0.2	0.67	-	-	-
1037	1037	Eucalyptol	35.63	34.67	34.48	0.1	0.23	0.59	tr	tr	tr
1061	1058	*γ*-Terpinene	4.21	5.16	4.15	tr	-	-	-	-	-
1104	1102	Linalool	1.36	0.81	1.64	87.88	86.46	77.58	tr	tr	tr
1108	1108	cis-*β*-Terpineol	0.57	0.44	0.54	-	-	-	-	-	-
1148	1139	Pinocarveol	1.93	1.92	2.09	-	-	-	-	-	-
1154	1150	Camphor	-	-	0.1	1.13	tr	7.85	97.78	92.47	97.04
1178	1172	Borneol	0.25	0.71	0.57	tr	0.55	0.37	1.23	-	1.28
1185	1176	4-Terpineol	6.18	8.53	7.44	tr	tr	0.23	-	-	-
1198	1193	*α*-Terpineol	19.75	19.15	20.96	0.35	0.6	0.89	-	-	-
1213	1207	Piperitol	0.16	0.15	0.16	-	-	-	-	-	-
1226	1223	*β*-Citronellol	0.32	0.35	0.34	-	-	-	-	-	-
1318	1318	Pinanediol	0.18	0.14	-	-	tr	0.15	-	-	-
Sesquiterpenes			5.64	4.83	9.27	8.41	10.45	8.44	0.00	3.64	0.00
1430	1418	*β*-caryophyllene	2.29	4.17	3.41	4.92	2.33	1.62	-	-	-
1466	1454	*α*-caryophyllene	0.35	0.5	0.4	0.69	0.92	0.63	-	-	-
1471	1461	Alloaromadendrene	-	-	0.19	0.13	0.21	-	-	-	-
1491	1484	Germacrene D	2.39	-	1.2	0.44	3.82	1.09	-	-	-
1505	1503	*β*-Chamigrene	-	0.16	0.25	0.11	2.78	1.06	-	-	-
1562	1563	trans-Nerolidol	0.61	-	3.82	1.58	0.29	2.25	-	-	-
1588	1577	(-)-Spathulenol	-	-	-	tr	tr	0.3	-	-	-
1594	1596	Caryophyllene oxide	-	-	-	0.17	-	-	-	1.91	-
1665	1653	*α*-Eudesmol	-	-	-	-	0.1	1.24	-	-	-
1786	1762	Aristolone	-	-	-	0.37		0.25	-	-	-
Phenols			0.19	0.00	0.00	0.00	0.35	0.18	0.00	0.00	0.00
1398	1404	Methyleugenol	0.19	-	-	tr	0.2	0.18	-	-	-
1495	1491	Methylisoeugenol	-	-	-	tr	0.15	-	-	-	-
Total %			98.81	98.48	99.31	97.87	98.84	96.95	99.01	94.38	98.32
Oil yield %			1.50	1.65	1.15	1.90	2.14	1.86	0.61	0.78	0.96

RT: Retention time, RIa: Retention indices calculated against n-alkanes, RIb: Retention indices reported in the literature [37,38,39,40]. tr, trace (<0.1%); ‘-’means ‘not found’. Y_L1, Y_L2 and Y_L3 were eucalyptol chemotype; F_L1, F_L2 and F_L3 were linalool chemotype; N_L1, N_L2 and N_L3 were camphor chemotype. Quantification was done using the calibration curves from the analyses of following standards. β-pinene was the standard of alkenes; eucalyptol was the standard of alcohols; 2-Dodecanonell was the standard of ketones; eugenol was the standard of phenols.

**Table 2:** Summary of the final transcript sequences obtained after the elimination of redundancy.

Sample	Total Isoforms	Total Base (bp)	Mean Length (bp)	N50 (bp)
Total	104,062	306,337,921	2944	5449

**Table 3:** Statistics of CDS prediction results.

Software	Total Number	Total Length	Mean Length	N50	N70	N90	GC (%)
Blast	88763	66645204	750	1041	687	363	44.66
ESTScan	340	205098	603	729	501	336	43.75
Overall	89103	66850302	750	1038	687	363	44.66

**Table 4:** Summary of RNA-seq data from nine RNA libraries of eucalyptol chemotype, camphor chemotype and linalool chemotype of *C. porrectum.*

Sample	Total Raw Reads (M)	Total Clean Reads (M)	Total Clean Bases (Gb)	Clean Reads Q20 (%)	Clean Reads Q30 (%)	Total Mapping (%)
AYYS1	45.72	42.54	6.38	98.79	96.38	80.41
AYYS2	45.72	42.45	6.37	98.82	96.46	79.94
AYYS3	45.72	42.36	6.35	98.78	96.37	81.93
NZ1	47.35	43.01	6.45	98.68	96.07	78.49
NZ2	47.35	43.24	6.49	98.73	96.2	81.5
NZ3	47.35	42.95	6.44	98.63	95.93	78.7
YXFZC1	47.35	42.51	6.38	98.27	94.78	76.74
YXFZC2	47.35	42.5	6.38	98.29	94.82	73.63
YXFZC3	47.35	42.75	6.41	98.31	94.9	74.33
summary	421.26	384.31	57.65	/	/	/

AYYS1, AYYS2and AYYS3 were eucalyptol chemotype; NZ1, NZ2 and NZ3 were camphor chemotype; YXFZC1, YXFZC2 and YXFZC3 were linalool chemotype.

## Supplementary Materials

Supplementary materials can be found at https://www.mdpi.com/1422-0067/20/24/6230/s1.

## Abbreviations

bp	Base pairs (measuring unit)
CDS	Coding sequence
DEGs	Differentially expressed genes
DESeq	Differentially expressed sequence
DMAPP	Dimethylallyl diphosphate
FDR	False discovery rate
FPKM	Fragments per kilobase of exon per million fragments mapped
FPPS	Farnesyl diphosphate synthase
GC-MS	Gas chromatography-mass spectrometry
GGPS	Geranylgeranyl diphosphate synthase
GO	Gene ontology
GPS	Geranyl diphosphate synthase
IPP	Isopentenyl diphosphate
KEGG	Kyoto encyclopedia of genes and genomes
KO	KEGG ortholog database
KOG	EuKaryotic Orthologous Groups
MEP	2-C-methyl-D-erythritol 4phosphate
MVA	Mevalonate acid
NIST	National institute of standards and technology
Nr	NCBI non-redundant protein sequences
Nt	NCBI non-redundant nucleotide sequences
Pfam	Protein family
qRT-PCR	Quantitative reverse transcription PCR
ROI	Reads of insert
RNA-Seq	RNA sequencing
SMRT	Single molecule real-time
TP	Terpene synthases
