# Supplementary material for: Full-Length Transcriptome Sequencing and Different Chemotype Expression Profile Analysis of Genes Related to Monoterpenoid Biosynthesis in Cinnamomum porrectum
Source: Int J Mol Sci. 2019 Dec 10;20(24):6230. doi: 10.3390/ijms20246230 (PMC6941020; doi:10.3390/ijms20246230)
Supplement: Supplementary file 1 [file ijms-20-06230-s001.zip › Supplementary files++/Table S1.Summary of read clustering information of full-length transcriptome libraries.docx]

Table S1. Summary of read clustering information of full-length transcriptome libraries

| Sample | Library | Cluster type | Total isoforms | Total base(bp) | Mean Quality | Mean isoform  length(bp) | Mean Full length  coverage | Mean Non Full  length coverage |
| --- | --- | --- | --- | --- | --- | --- | --- | --- |
| H0 | r54040_20171112  _084809-2_D01 | High quality | 42681 | 1.25E+08 | 0.9983 | 2920 | 1.42 | 15.65 |
| H0 | r54040_20171112  _084809-2_D01 | Low quality | 20773 | 65017217 | 0.5634 | 3130 | 1.07 | 10.74 |
| H0 | r54160_20171114  _083712-1_F01 | High quality | 91285 | 2.71E+08 | 0.9984 | 2973 | 1.55 | 28.91 |
| H0 | r54160_20171114  _083712-1_F01 | Low quality | 58847 | 1.67E+08 | 0.5163 | 2841 | 1.1 | 16 |
