# Supplementary figures and images for: Full-Length Transcriptome Sequencing and Different Chemotype Expression Profile Analysis of Genes Related to Monoterpenoid Biosynthesis in Cinnamomum porrectum
Source: Int J Mol Sci. 2019 Dec 10;20(24):6230. doi: 10.3390/ijms20246230 (PMC6941020; doi:10.3390/ijms20246230)

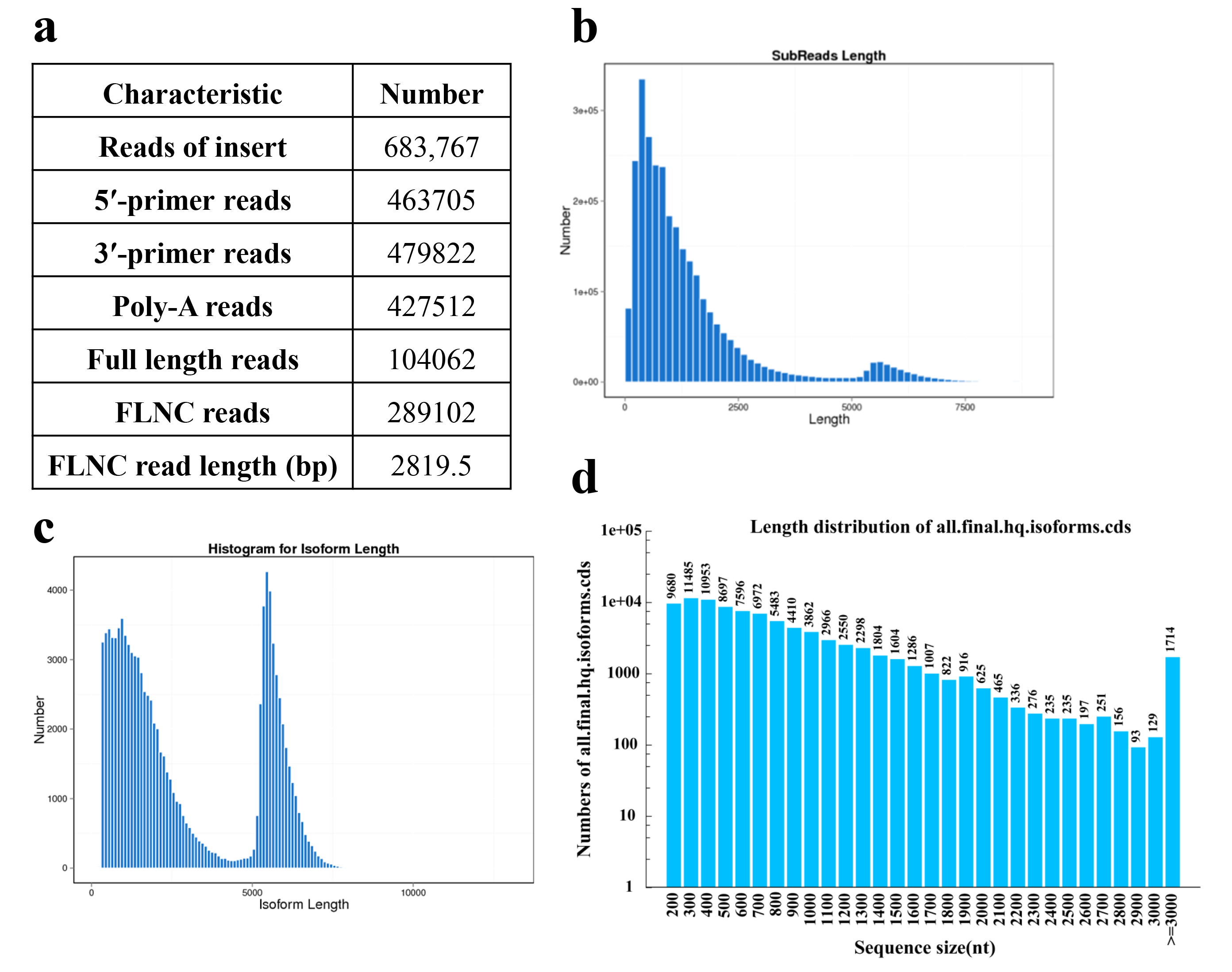

Supplement: Supplementary file 1 [file ijms-20-06230-s001.zip › Supplementary files++/Figures/Fig 1.tif]

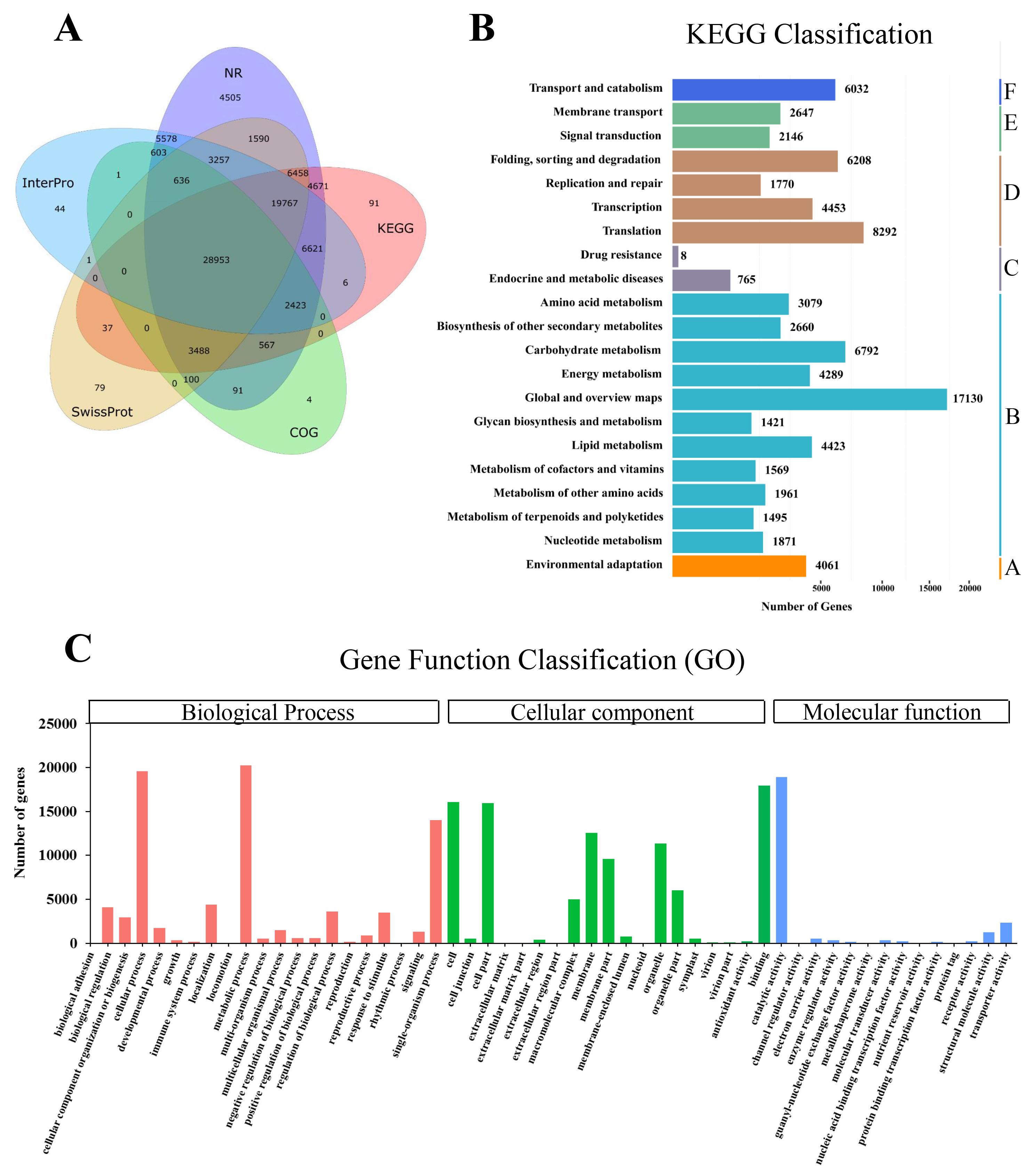

Supplement: Supplementary file 1 [file ijms-20-06230-s001.zip › Supplementary files++/Figures/Fig 2.tif]

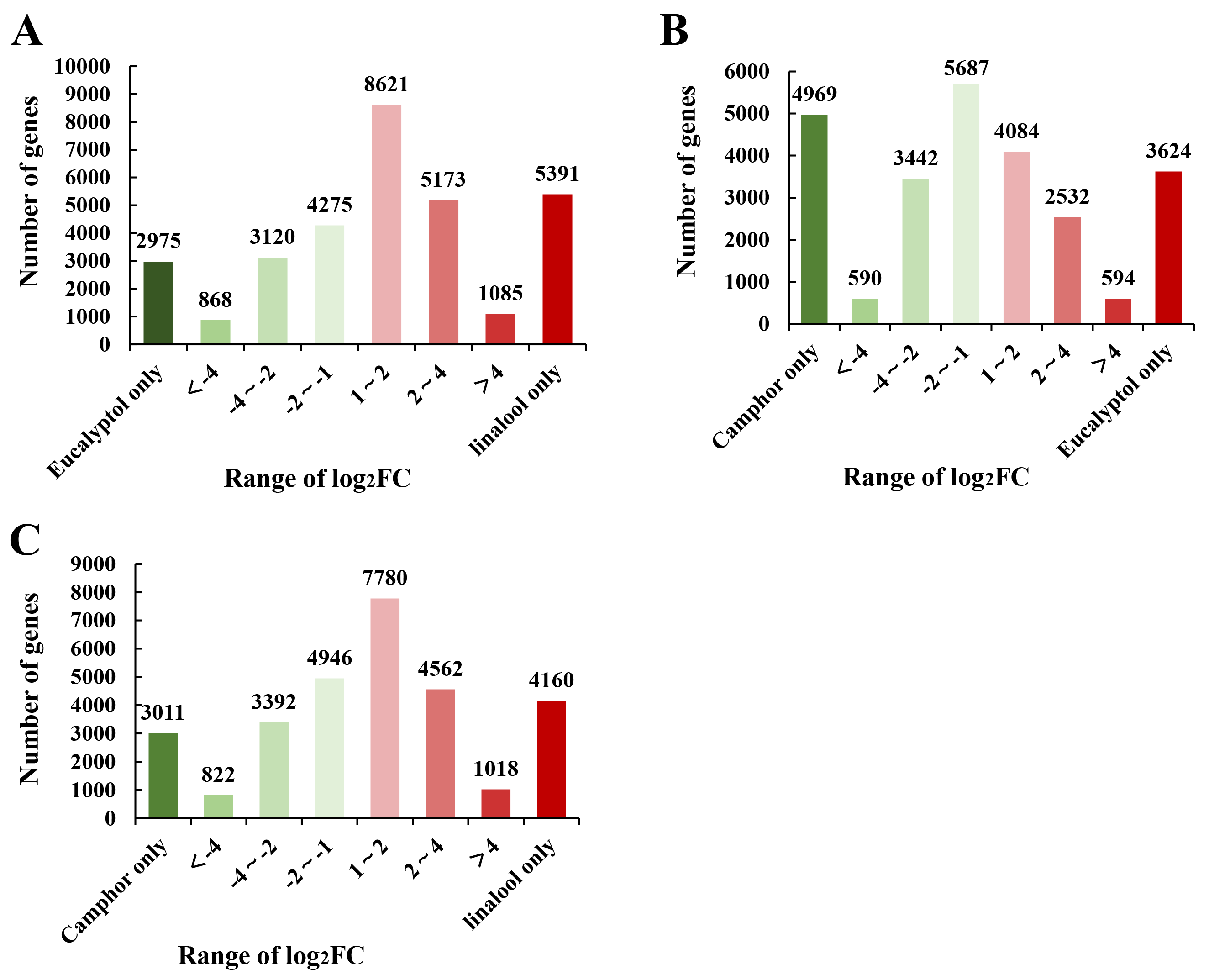

Supplement: Supplementary file 1 [file ijms-20-06230-s001.zip › Supplementary files++/Figures/Fig 3.tif]

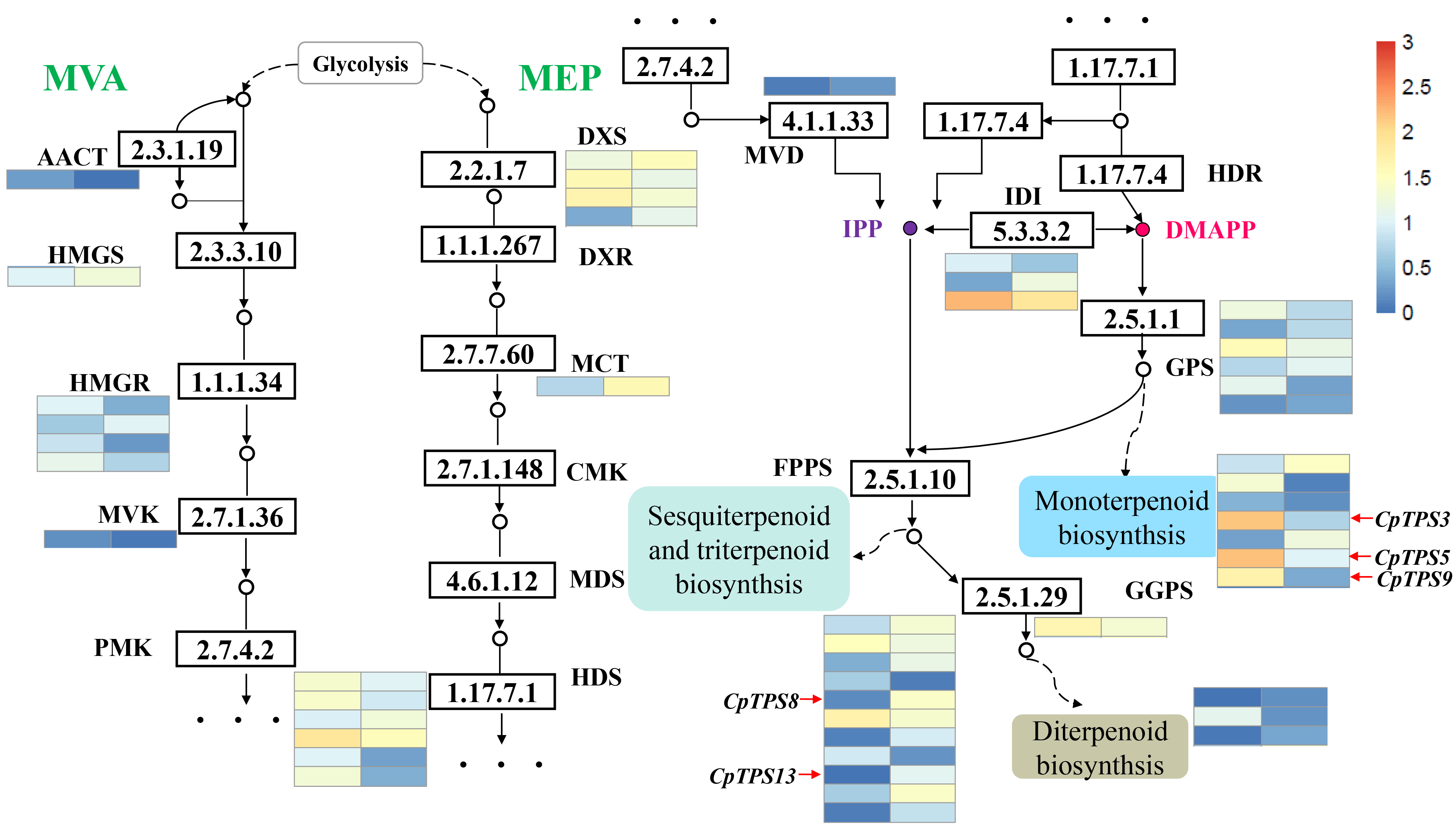

Supplement: Supplementary file 1 [file ijms-20-06230-s001.zip › Supplementary files++/Figures/Fig 4.tif]

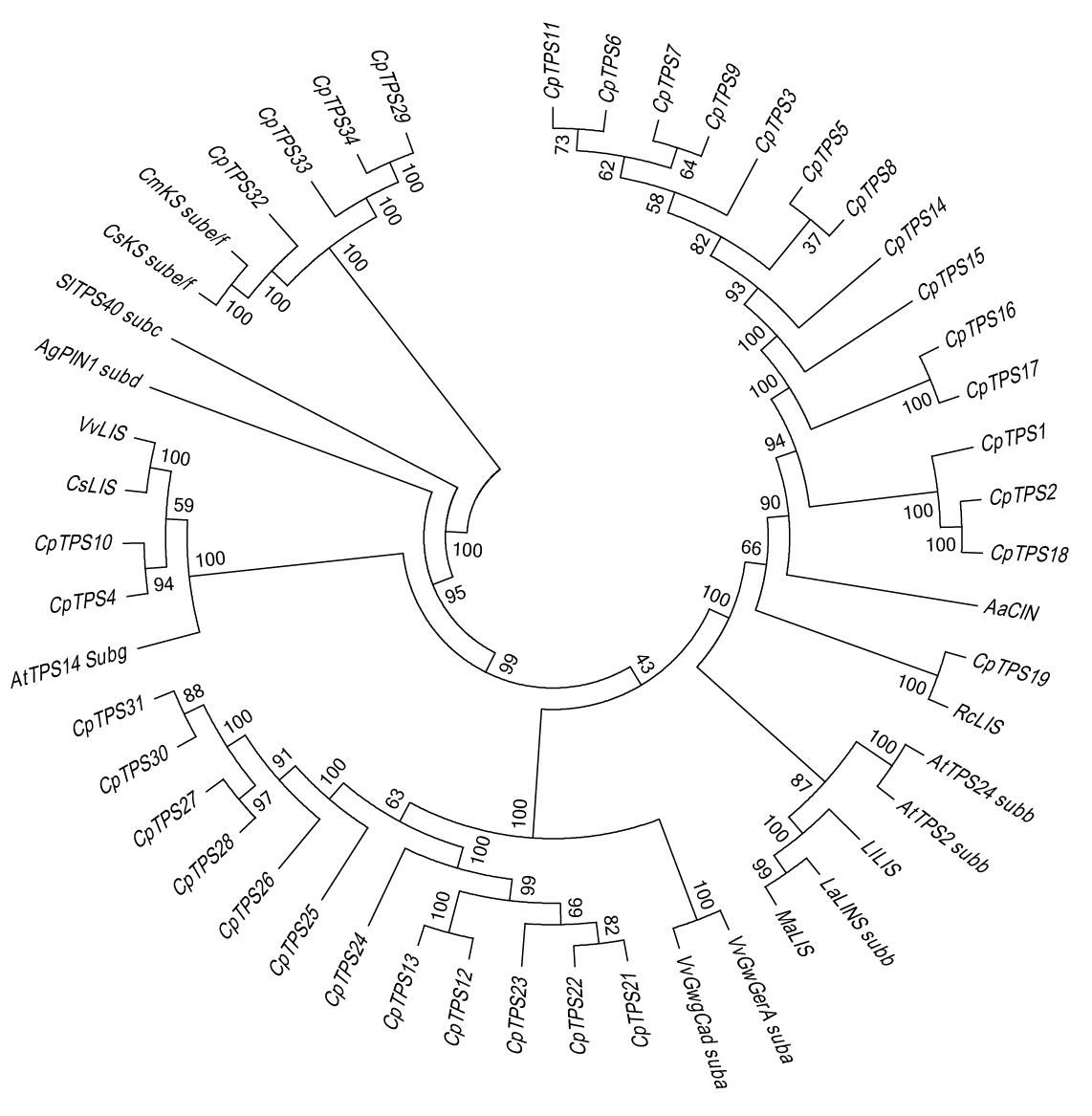

Supplement: Supplementary file 1 [file ijms-20-06230-s001.zip › Supplementary files++/Figures/Fig 5.tif]

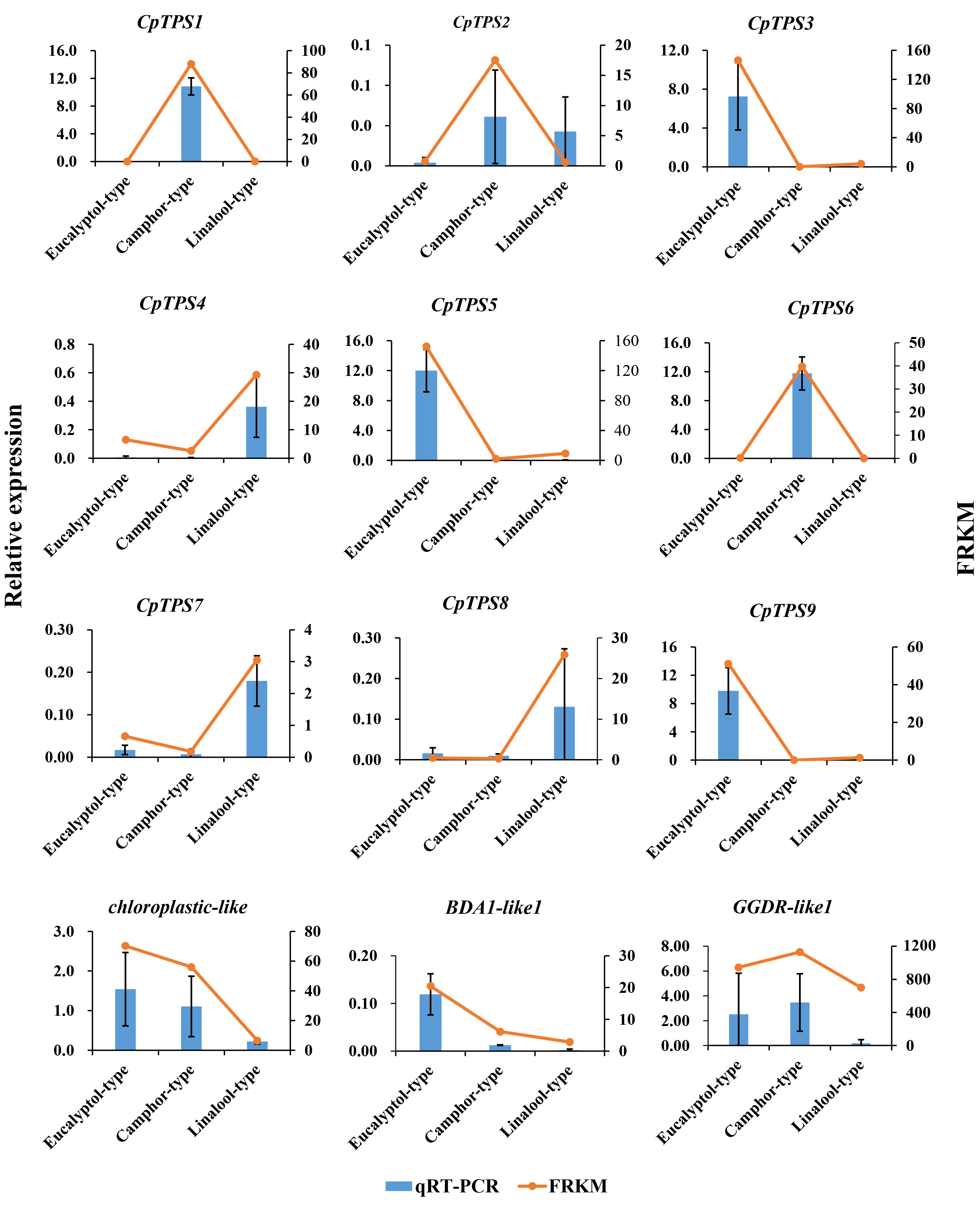

Supplement: Supplementary file 1 [file ijms-20-06230-s001.zip › Supplementary files++/Figures/Fig 6.tif]

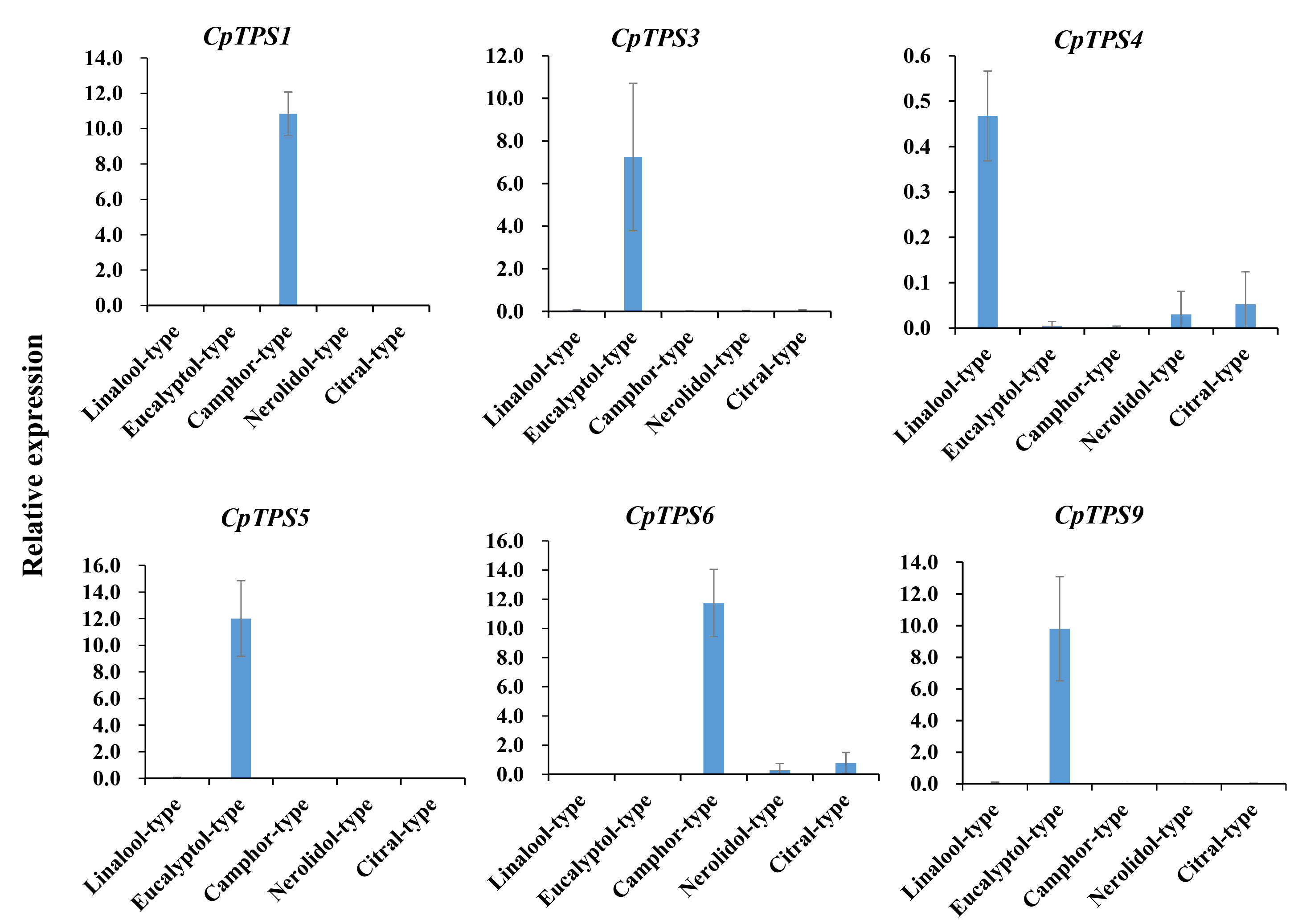

Supplement: Supplementary file 1 [file ijms-20-06230-s001.zip › Supplementary files++/Figures/Fig 7.tif]
