# Supplementary material for: Full-Length Transcriptome Sequencing and Different Chemotype Expression Profile Analysis of Genes Related to Monoterpenoid Biosynthesis in Cinnamomum porrectum
Source: Int J Mol Sci. 2019 Dec 10;20(24):6230. doi: 10.3390/ijms20246230 (PMC6941020; doi:10.3390/ijms20246230)
Supplement: Supplementary file 1 [file ijms-20-06230-s001.zip › Supplementary files++/Supplemental materials-Figure S1-S4.doc]

**Supplemental materials**

**Figure S1.** The sequence length and sequence quality of the low-QV and high-QV sequences

**Figure S2.** Dot plot of the top 20 KEGG pathway categories of DEGs in different chemotypes comparison groups of *C. porrectum* . (A) Dot plot of the top 20 KEGG pathway categories of DEGs in the eucalyptol and linalool chemotype comparison group; (B) Dot plot of the top 20 KEGG pathway categories of DEGs in the camphor and eucalyptol chemotype comparison group; (C)Dot plot of the top 20 KEGG pathway categories of DEGs in the camphor and linalool chemotype comparison group.

**Figure S3.** DEGs in terpenoid backbone biosynthesis in the camphor chemotype and eucalyptol chemotype comparison group of *C. porrectum*. Terpenoids biosynthesis pathway showing the subset of nodes or enzymes that constitute the process. Enzymes expression patterns are indicated at the side of each step with the value of log(FPKM+ 1). The expression pattern of each unigene is shown within two-column grids, with the left column representing the camphor chemotype and the right one representing the eucalyptol chemotype. Red means high expression and blue means low expression, the color gradually from blue to red represents gene expression abundance from low to high. CCAT, Acetyl-CoA C-acetyltransferase; HMGS, hydroxymethylglutaryl-CoA synthase; HMGR, hydroxymethylglutaryl-CoA reductase (NADPH); MVK, mevalonate kinase; PMK, phosphomevalonate kinase; MVD, diphosphomevalonate decarboxylase; DXS, 1-deoxy-D-xylulose-5-phosphate synthase; DXR, 1-deoxy-Dxylulose-5-phosphate reductoisomerase; MCT, 2-C-methyl-D-erythritol 4-phosphate cytidylyltransferase; CMK, 4-diphosphocytidyl-2-C-methyl-D-erythritol kinase; MDS, 2-C-methyl-D-erythritol 2,4-cyclodiphosphate synthase; HDS, (E)-4-hydroxy-3-methylbut-2-enyl-diphosphate synthase; HDR, 4-hydroxy-3-methylbut-2-en-1-yl diphosphate reductase; IDI, isopentenyl-diphosphate Delta-isomerase; DMAPP, dimethylallyl diphosphate; GPS, geranyl diphosphate synthase; FPPS, farnesyl diphosphate synthase; GGPS, geranylgeranyl diphosphate synthase

**Figure S4.** DEGs in terpenoid backbone biosynthesis in the camphor chemotype and linalool chemotype comparison group of *C. porrectum*. Terpenoids biosynthesis pathway showing the subset of nodes or enzymes that constitute the process. Enzymes expression patterns are indicated at the side of each step with the value of log(FPKM+ 1). The expression pattern of each unigene is shown within two-column grids, with the left column representing the camphor chemotype and the right one representing the linalool chemotype. Red means high expression and blue means low expression, the color gradually from blue to red represents gene expression abundance from low to high. CCAT, Acetyl-CoA C-acetyltransferase; HMGS, hydroxymethylglutaryl-CoA synthase; HMGR, hydroxymethylglutaryl-CoA reductase (NADPH); MVK, mevalonate kinase; PMK, phosphomevalonate kinase; MVD, diphosphomevalonate decarboxylase; DXS, 1-deoxy-D-xylulose-5-phosphate synthase; DXR, 1-deoxy-Dxylulose-5-phosphate reductoisomerase; MCT, 2-C-methyl-D-erythritol 4-phosphate cytidylyltransferase; CMK, 4-diphosphocytidyl-2-C-methyl-D-erythritol kinase; MDS, 2-C-methyl-D-erythritol 2,4-cyclodiphosphate synthase; HDS, (E)-4-hydroxy-3-methylbut-2-enyl-diphosphate synthase; HDR, 4-hydroxy-3-methylbut-2-en-1-yl diphosphate reductase; IDI, isopentenyl-diphosphate Delta-isomerase; DMAPP, dimethylallyl diphosphate; GPS, geranyl diphosphate synthase; FPPS, farnesyl diphosphate synthase; GGPS, geranylgeranyl diphosphate synthase
