# Supplementary material for: Full-Length Transcriptome Sequencing and Different Chemotype Expression Profile Analysis of Genes Related to Monoterpenoid Biosynthesis in Cinnamomum porrectum
Source: Int J Mol Sci. 2019 Dec 10;20(24):6230. doi: 10.3390/ijms20246230 (PMC6941020; doi:10.3390/ijms20246230)
Supplement: Supplementary file 1 [file ijms-20-06230-s001.zip › Supplementary files++/Table S2. Summary of gene annotation by seven databases.doc]

Table S2. Summary of gene annotation by seven databases

| Values | Total | Nr-Annotated | Nt-Annotated | Swissprot-Annotated | KEGG-Annotated | COG-Annotated | Interpro-Annotated | GO-Annotated | Intersection | Overall |
| --- | --- | --- | --- | --- | --- | --- | --- | --- | --- | --- |
| Number | 104,062 | 89,308 | 84,487 | 64,366 | 73,082 | 36,866 | 67,890 | 35,698 | 21.781 | 94,025 |
| Percentage | 100% | 85.82% | 81.19% | 61.85% | 70.23% | 35.43% | 65.24% | 34.26% | 20.93% | 90.35% |
