# Supplementary material for: Full-Length Transcriptome Sequencing and Different Chemotype Expression Profile Analysis of Genes Related to Monoterpenoid Biosynthesis in Cinnamomum porrectum
Source: Int J Mol Sci. 2019 Dec 10;20(24):6230. doi: 10.3390/ijms20246230 (PMC6941020; doi:10.3390/ijms20246230)
Supplement: Supplementary file 1 [file ijms-20-06230-s001.zip › Supplementary files++/Table S5. Primers sequences used for qRT-PCR.doc]

Table S5. Primers sequences used for *q*RT-PCR

| Primers name | Forward primer sequence | Reverse primer sequence |
| --- | --- | --- |
| *Cp TPS1*-qPCR | GAACTAAAAGCCCAGCATCT | CGAAGAGCCACCTTAGACC |
| *Cp TPS2*-qPCR | ACGGGCACAAGGTCTCGG | GCTCTTGCCTCATCCAAGAT |
| *Cp TPS3*-qPCR | AAAAGGATGTCAAGGTGGTG | CGCTCAAACTGTGGGTCA |
| *Cp TPS4*-qPCR | ACCACCCAAGACCCTGAC | CTACTGCCATTGCCACCA |
| *Cp TPS5*-qPCR | AGGATGTCAAGGTGGTGGAG | CCAACTCCTCCAAAGAACCA |
| *Cp TPS6*-qPCR | GGAACTACAAGCCCAGCATC | TCATTTTCGAAGAGCCACCT |
| *Cp TPS7*-qPCR | TGGATGGAATCCCATTGACT | AACGATATGCACTCCGGAAC |
| *Cp TPS8*-qPCR | GTTCCGGAGTGCATATCGTT | GGTCCCATGCTCTTTCATGT |
| *Cp TPS9*-qPCR | AGGATGTCAAGGTGGTGGAG | CCAACTCCTCCAAAGAACCA |
| *Chloroplastic-like*-qPCR | TCCTCTGCTTACCACCATCC | ACGTTCTCGTCCAGATCCAC |
| *BDA1-like1-*qPCR | TTCAGCGCAAGGTCATGTAG | AGTGGCTCTCCTGCATCAGT |
| *GGDR-like1*-qPCR | AGGTGTGCAGAAGGCAGACT | ATGGCATACACCCCATAGGA |
| *Actin-*qPCR | CCTCGACACACAGGCGTTAT | CCATGCTCGATGGGATATTTCA |
