# Supplementary material for: Full-Length Transcriptome Sequencing and Different Chemotype Expression Profile Analysis of Genes Related to Monoterpenoid Biosynthesis in Cinnamomum porrectum
Source: Int J Mol Sci. 2019 Dec 10;20(24):6230. doi: 10.3390/ijms20246230 (PMC6941020; doi:10.3390/ijms20246230)
Supplement: Supplementary file 1 [file ijms-20-06230-s001.zip › Supplementary files++/Table S9. Composition of the leaf extracts of nerolidol-type and citral-type.doc]

Table S9 Composition of the leaf extracts of nerolidol-type and citral-type

| RIa | RI_b_ | Component | Nerolidol-type-1  (%) | Nerolidol-type-2  (%) | Nerolidol-type-3  (%) | Citral-type-1  (%) | Citral-type-2  (%) | Citral-type-3  (%) |
| --- | --- | --- | --- | --- | --- | --- | --- | --- |
| 986 | 978 | beta.-Pinene | - | - | - | 0.19 | - | 0.20 |
| 1104 | 1102 | Linalool | 2.14 | 0.39 | 0.97 | 2.22 | 2.34 | 3.11 |
| 1143 | 1150 | 2,5-dimethyl-1,6-Octadiene | - | - | - | 1.45 | 1.44 | 1.52 |
| 1154 | 1150 | Camphor | 0.10 | 0.67 | 0.40 | 2.85 | 4.08 | - |
| 1161 |  | Berbenol | - | - | - | 3.63 | 3.43 | 3.96 |
| 1178 | 1172 | Borneol | 0.93 | - | 0.37 | - | - | - |
| 1180 | 1173 | 1,3,4-trimethyl-3-Cyclohexene-1-carboxaldehyde | - | - | - | 4.91 | 4.82 | 5.37 |
| 1185 | 1176 | 4-Terpineol | 0.10 | - | - | 0.31 | 0.32 | 0.46 |
| 1198 | 1193 | L-alpha-Terpineol | 0.64 | - | 0.27 | 0.57 | 0.48 | 0.75 |
| 1226 | 1223 | beta-Citronellol | - | - | - | 2.45 | 2.63 | 2.08 |
| 1239 | 1244 | beta-Citral | - | - | - | 23.72 | 23.05 | 24.77 |
| 1249 | 1249 | Geraniol | 0.16 | - | - | 5.27 | 3.27 | 4.22 |
| 1270 | 1273 | alpha.-Citral | - | - | - | 29.39 | 28.74 | 31.88 |
| 1342 | 1337 | cis-Verbenol | 0.10 | 0.31 | 0.19 | - | - | - |
| 1398 | 1404 | Methyleugenol | 0.25 | 0.77 | 0.37 | - | 0.28 | - |
| 1430 | 1418 | beta-caryophyllene | 7.62 | 6.97 | 7.11 | 2.87 | 4.17 | 3.36 |
| 1466 | 1454 | alpha-caryophyllene | 1.31 | 4.43 | 3.15 | 0.41 | 0.53 | 0.30 |
| 1471 | 1461 | Alloaromadendrene | 1.15 | - | 0.51 | 0.15 | 0.32 | 0.29 |
| 1491 | 1484 | Germacrene D | 4.99 | 9.85 | 7.94 | 0.20 | 0.96 | 1.00 |
| 1495 | 1491 | Methylisoeugenol | 0.11 | 0.78 | 0.55 | - | - | - |
| 1505 | 1503 | beta-Chamigrene | 0.56 | 2.44 | 1.60 | - | - | - |
| 1556 | 1549 | Elemol | - | - | - | 6.06 | - | - |
| 1562 | 1563 | trans-Nerolidol | 77.48 | 52.55 | 64.93 | - | 5.08 | 4.48 |
| 1588 | 1577 | (-)-Spathulenol | - | 0.62 | 0.51 | - | - | - |
| 1594 | 1596 | Caryophyllene oxide | - | - | - | 0.26 | 1.14 | 0.54 |
| 1634 | 1615 | Selina-6-en-4-ol | tr | 0.20 | - | 0.18 | - | - |
| 1643 | 1636 | gama-eudesmol | - | 0.20 | 0.29 | 2.04 | - | - |
| 1665 | 1652 | alpha-Eudesmol | 0.15 | 0.22 | 0.26 | 3.99 | 0.83 | 0.81 |
| 1686 |  | Aristolone | - | 2.19 | 1.30 | - | 0.83 | 0.45 |
| 1717 | 1714 | Farnesol | 0.69 | 0.73 | 0.33 | - | - | - |
| 1729 |  | 3-Methyl-but-2-enoic acid isoborneol ester | - | 11.51 | 5.97 | - | 4.47 | 2.69 |
| 1830 |  | 4-Hydroxy-beta-ionone | - | 0.51 | 0.36 | - | - | - |
| 2035 |  | Terpinyl isobutyrate | - | - | - | 1.18 | 0.80 | 0.90 |
| 2093 |  | trans-Dihydrocarvyl acetat | - | - | - | 5.00 | 4.02 | 4.44 |
| Total （%） | | | 98.48 | 95.34 | 97.38 | 97.85 | 96.59 | 96.06 |

RT:Retention time, RIa :Retention indices calculated against n-alkanes, RI_b_: Retention indices reported in the literature. tr, trace(＜0.1%),‘-’means ‘not found’. Quantification was done using the calibration curves from the analyses of following standards. β-pinene was the standard of alkenes; eucalyptol was the standard of alcohols, 2-Dodecanonell was the standard of ketones; eugenol was the standard of phenols, tetradecanalwas the standard of aldehyde, methyl palmitate was the standard of ester.
